# Economic benefit analysis of lithium battery recycling based on machine learning algorithm

**DOI:** 10.1371/journal.pone.0303933

**Published:** 2024-06-07

**Authors:** Jie Zhang

**Affiliations:** School of Accounting, Xijing University, Xi’an, China; Hanyang University - Seoul Campus: Hanyang University, REPUBLIC OF KOREA

## Abstract

Lithium batteries, as an important energy storage device, are widely used in the fields of renewable vehicles and renewable energy. The related lithium battery recycling industry has also ushered in a golden period of development. However, the high cost of lithium battery recycling makes it difficult to accurately evaluate its recycling value, which seriously restricts the development of the industry. To address the above issues, machine learning will be applied in the field of economic benefit analysis for lithium battery recycling, and backpropagation neural networks will be combined with stepwise regression. On the basis of considering social and commercial values, a lithium battery recycling and utilization economic benefit analysis model based on stepwise regression backpropagation neural network was designed. The experimental results show that the mean square error of the model converges between 10–6 and 10–7, and the convergence speed is improved by 33%. In addition, in practical experiments, the model predicted the actual economic benefits of recycling a batch of lithium batteries. The results show that the predictions are basically in line with the true values. Therefore, the economic benefit analysis and prediction model for lithium battery recycling proposed in the study has the advantages of high accuracy and fast operation speed, providing new ideas and tools for promoting innovation in the field of economic benefit analysis. It has certain application potential in the evaluation of the benefits of lithium battery recycling.

## 1. Introduction

Lithium batteries, as the core energy storage technology in the field of new energy, have been widely applied and promoted. However, as the quick advancement of the number of lithium batteries, the treatment and recycling of battery waste has become an important environmental and economic challenge. Experts in relevant fields have conducted extensive research on this issue. However, traditional financial models were often based on linear assumptions and statistical methods, which cannot capture complex nonlinear relationships and market dynamics. With the increasing application of machine learning (ML) algorithms in various industries, in-depth analysis of their economic benefits has become an important research field. ML algorithms, as a technology that utilizes computer automatic learning and improvement, can discover hidden patterns and rules from a large amount of data, providing strong support for decision-making [1, 2]. By analyzing and modeling data from fresh and recycled lithium batteries, ML models can predict the remaining capacity of recycled batteries, providing guidance for the reuse of recycled batteries. This predictive ability helps to optimize the secondary utilization of batteries, reduce the use of unqualified batteries and production costs [3]. Guided by the social and commercial value of lithium battery recycling (LBR), the research solves the multicollinearity problem between evaluation indicators based on BP neural network (BP) and stepwise regression (SR), and designs a stepwise regression BP neural network (SR-BPNN). It hopes that this study can provide a more accurate and stable analysis plan in the field of economic benefit analysis (EBA). The research mainly contains four parts. The second part summarizes the current research condition in the field of EBA, and summarizes the research achievements and methods all over world. In the third part, the BP is improved to design an EBA model of LBR based on SR-BPNN. In the fourth part, the optimization effect of the model is evaluated through comparative experiments and efficiency verification, and a horizontal comparison of the algorithms is organized to assess the accuracy and other performance aspects of the algorithm. The final part is a summary and discussion of the entire content.

## 2. Related works

As the widespread application of ML and other technologies in the economic field, more accurate and fast EBA models are gradually receiving attention from more enterprises and researchers. To solve the problem that the coupling of multiphysics simulation makes the performance of SOFC difficult to predict, researchers such as Song S designed a performance prediction model based on BP SOFC. The experiment outcomes denoted that the prediction accuracy, generalization ability and test time of BP were optimal [4]. Mukkamala R et al. designed a cuff free BP measurement device to address the issue of low accuracy in blood pressure measurement without a cuff device. The research findings expressed that the accuracy of the cuff BP measurement device has been improved by 8.2% [5]. To investigate the impact of antihypertensive therapy on the risk of major cardiovascular events through baseline systolic blood pressure, researchers such as Rahimi K established a cardiovascular risk prediction scheme based on the BP algorithm. The research findings showed that the accuracy of this scheme was as high as 98% [6]. Wright L G et al. proposed a method for offline signature recognition calculation using BP system and image processing technology to raise the accuracy of signature recognition. The experiment outcomes indicated that this method had a high success rate [7]. Zhang M et al. put forward a BP learning algorithm with spike timing to address the issue of using spatiotemporal pulse patterns to represent and transmit information in pulse neural networks that does not conform to biological reality. The experiment findings illustrated that the neural morphology hardware consumed a total power consumption of 0.751 mW and achieved a low latency of 47.71 ms [8]. Scholars such as Aswathy A L have designed a disease classification model that combines ResNet-50, DenseNet-201, and BPs for computer-aided diagnosis and severity detection. The findings expressed that the classification accuracy of this method was as high as 98.5% [9].

The research of ML in the economic field is deepening, which provides a certain reference for the EBA model of LBR. Sindhu J et al. designed a ML based investment return analysis model for commercial banks to address the issue of hard to accurately estimate the economic benefits of commercial banks. The experiment outcomes illustrated that the model had high accuracy and generalization ability [10]. Ibrahim A and other researchers designed a minimum maximum simple cost-benefit analysis (CBA) matrix to obtain executives’ views on using two segmentation methods (quantitative and qualitative). The research outcomes expressed that the financial costs of the quantitative segmentation method were higher than those of the qualitative method [11]. Cronin P et al. evaluated the economic benefits brought by rehabilitation colleges by analyzing the health services provided by service users before and after use, and then calculated the net cost savings. The outcomes denoted that after participating in rehabilitation colleges, there was a statistically significant decrease in the utilization rate of mental health for emergency departments and inpatients [12]. Szymanski T and other researchers designed a risk prediction analysis of algorithms method based on ML to improve the accuracy of using atrial fibrillation risk prediction algorithm. The experiment findings indicated that this method could reduce the detection error of atrial fibrillation and prevent stroke [13]. Boltürk E et al. proposed a decomposed fuzzy linguistic term scale to compare the costs required to achieve products, services or results with the benefits to be obtained, and developed new equations for CBA under fuzziness. The findings denoted that this method was conducive to the successful application of engineering economics and financial analysis methods under fuzziness [14]. Ward ZJ and other researchers evaluated the investment costs and benefits of improving cancer survival by evaluating a package of treatments, imaging methods, and quality of care. The findings expressed that expanding treatment and quality of care without imaging would result in a return of $6.15 per dollar investment [15].

Sheikh et al. proposed a method for intelligent analysis of battery status using machine learning algorithms to address the issues of battery performance monitoring and management. This method estimates battery performance parameters by extracting discharge curve features. The results show that this method can effectively evaluate the battery status under different current and temperature conditions, providing a new approach for battery management [16]. M. Khalid et al. proposed a SOC evaluation method based on the Thevenin equivalent circuit model for lithium-ion battery state estimation in electric vehicles, using Coulomb counting method and Extended Kalman Filter (EKF). The results showed that compared to Coulomb counting, using EKF to evaluate SOC was more accurate, with an error reduction of 1% [17]. Sheikh and other researchers proposed three data-driven methods for lithium-ion battery condition monitoring and capacity estimation, namely convolutional neural networks, feedforward neural networks, and long short-term memory networks, for comparison. The results show that machine learning techniques based on long short-term memory networks have excellent performance and high accuracy, and it is recommended for researchers to use them [18]. Shah et al. proposed a review of the latest battery health assessment technology on the application and related issues of lithium-ion batteries in electric and hybrid vehicles. The results show that there are differences in models/algorithms, errors, advantages and disadvantages, and system costs among different technologies, with some performing better [19].

In summary, the application of ML in the field of EBA has sufficient theoretical and implementation foundations, but traditional EBA models cannot meet the accuracy needs of users well. Therefore, on the basis of BP, combined with SR, the research establishes an EBA model with higher accuracy and stronger generalization ability to promote the further development of the LBR industry.

## 3. Design of an EBA model based on ML

This chapter is divided into three sub-sections. The first section first constructs the evaluation system related to the economic benefits of LBR, and divides the economic benefits into social and commercial values. The second section designed an economic benefit evaluation model based on BP, which preliminarily achieved accurate prediction of economic benefits. The third section improves the BP by combining SR with it, further improving the accuracy of the prediction model.

### 3.1 Construction of economic benefit evaluation system for LBR

**Fig 1:**
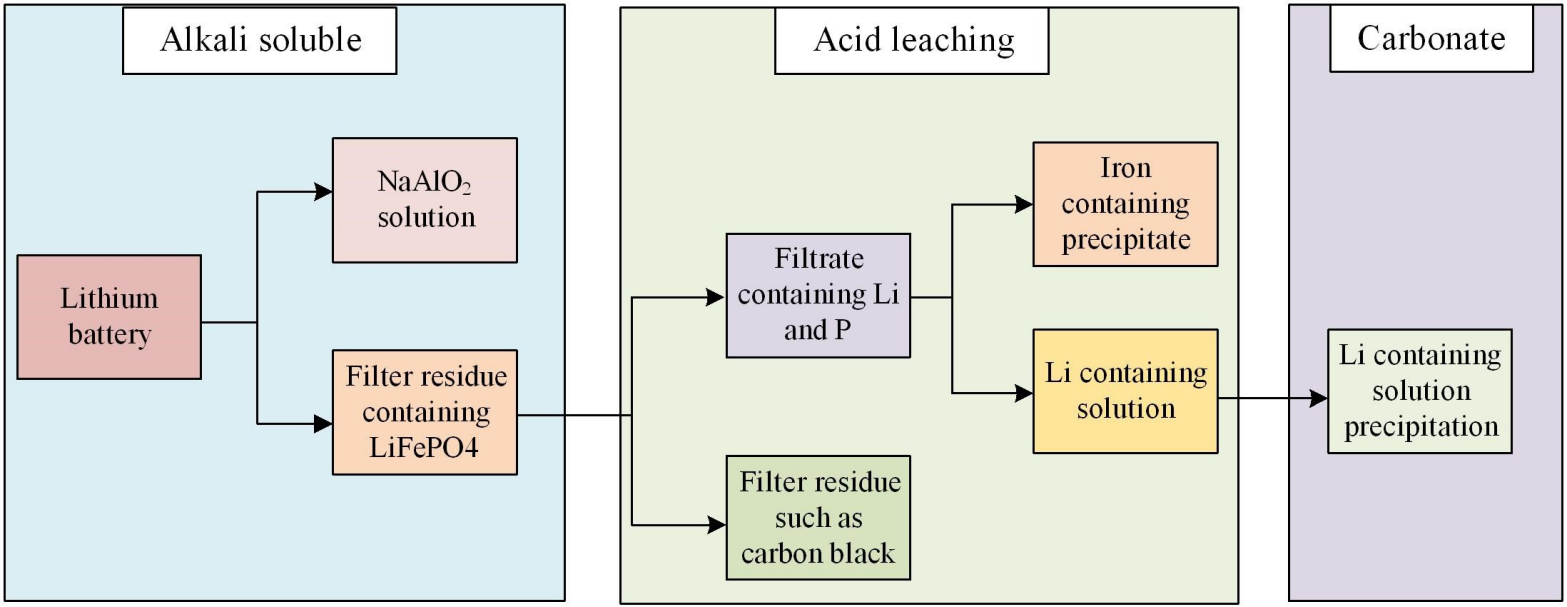
Schematic diagram of hydrometallurgy method.

LBR is a rather complicated process. There are many kinds of technologies. Taking hydrometallurgy as an example, it is a common method for recovering and extracting valuable materials from Lithium iron phosphate (LIP) batteries [20]. The following is a typical hydrometallurgical process for recovering LIP battery. First, the waste LIP battery needs to be pretreated, including removing the shell and other non-valuable components, such as electrolyte, diaphragm, etc. After pre-treatment, waste batteries enter the crushing stage. This step decomposes the battery into small pieces or particles for subsequent processing. In the extraction stage, chemical reaction or physical separation technology is used to extract LIP compound from crushed waste batteries [21]. This may involve leaching, dissolution, precipitation, filtration and other steps to separate LIP. The extracted LIP compound usually needs further treatment and purification to remove impurities and improve the purity of the product. This may include operations such as dissolution, filtration, crystallization, washing, etc. Drying and crushing of materials: after treatment and purification, the obtained LIP material needs to be dried to remove water, and necessary crushing or refining treatment is required to obtain the required product morphology and granularity [22]. Finally, the LIP recovered through the hydrometallurgical process can be re-supplied to the battery manufacturing industry to produce new LIP battery as raw materials, to realize the reuse of resources. It can also be sold to other related industries as valuable materials, as shown in Fig 1.

**Fig 2:**
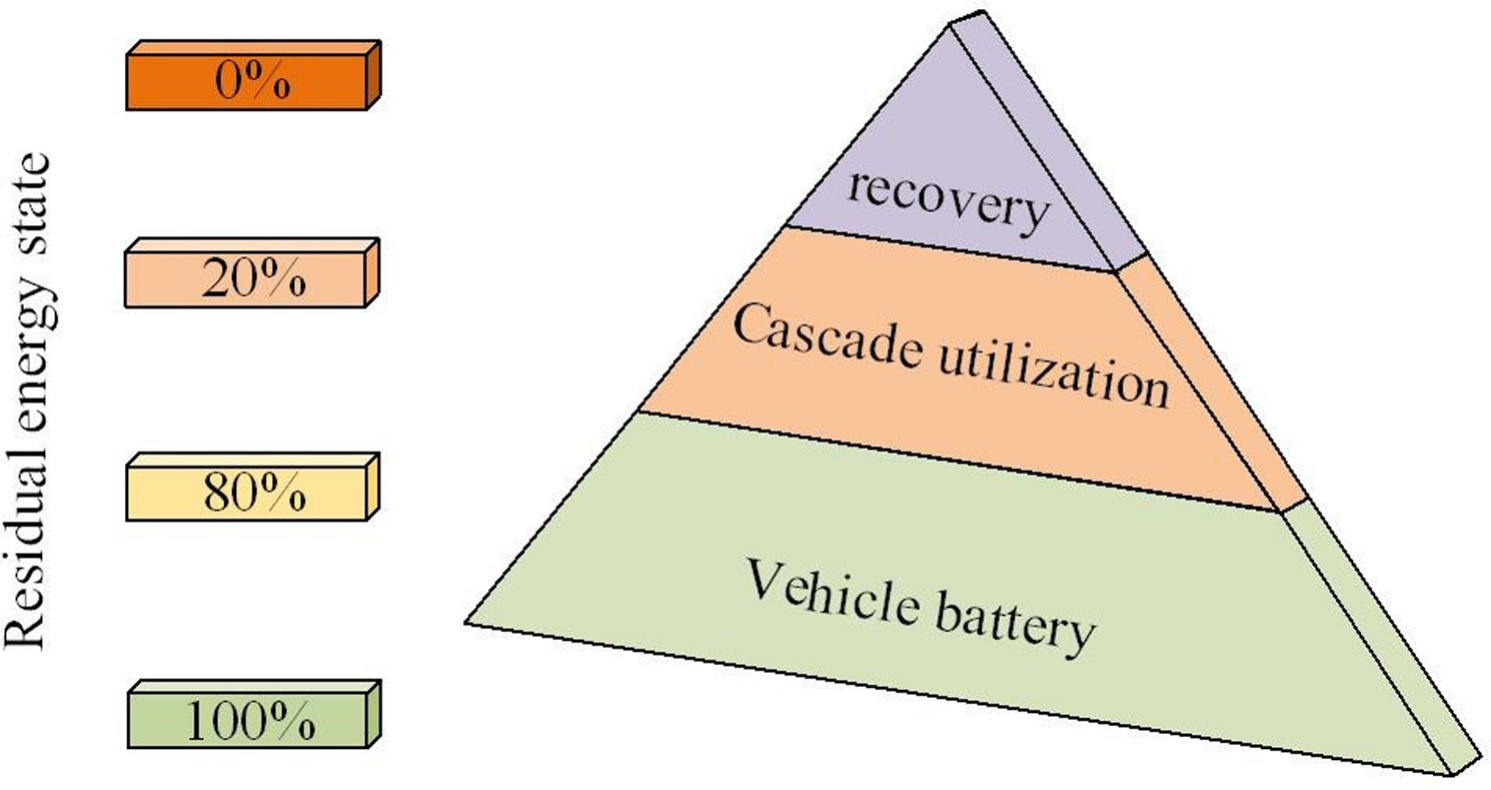
Schematic diagram of cascade utilization.

The key factors that determine whether chemical power sources can benefit economically from energy storage are cost and cycle life. Along the usage time rises, the internal capacity of the power battery will gradually lessen, and the battery performance will decrease. When the internal capacity of the power battery of new energy vehicles decreases to the point where it cannot meet the requirements of the original level of vehicles, it will face scrapping. However, scrapped batteries can usually serve as backup batteries to power some low energy consuming products, or used for energy storage in clean renewable energy power stations and telecommunications base stations. The cascade utilization of power batteries for energy storage fundamentally solves the problem of uneven distribution of energy in time and space, as shown in Fig 2.

**Fig 3:**
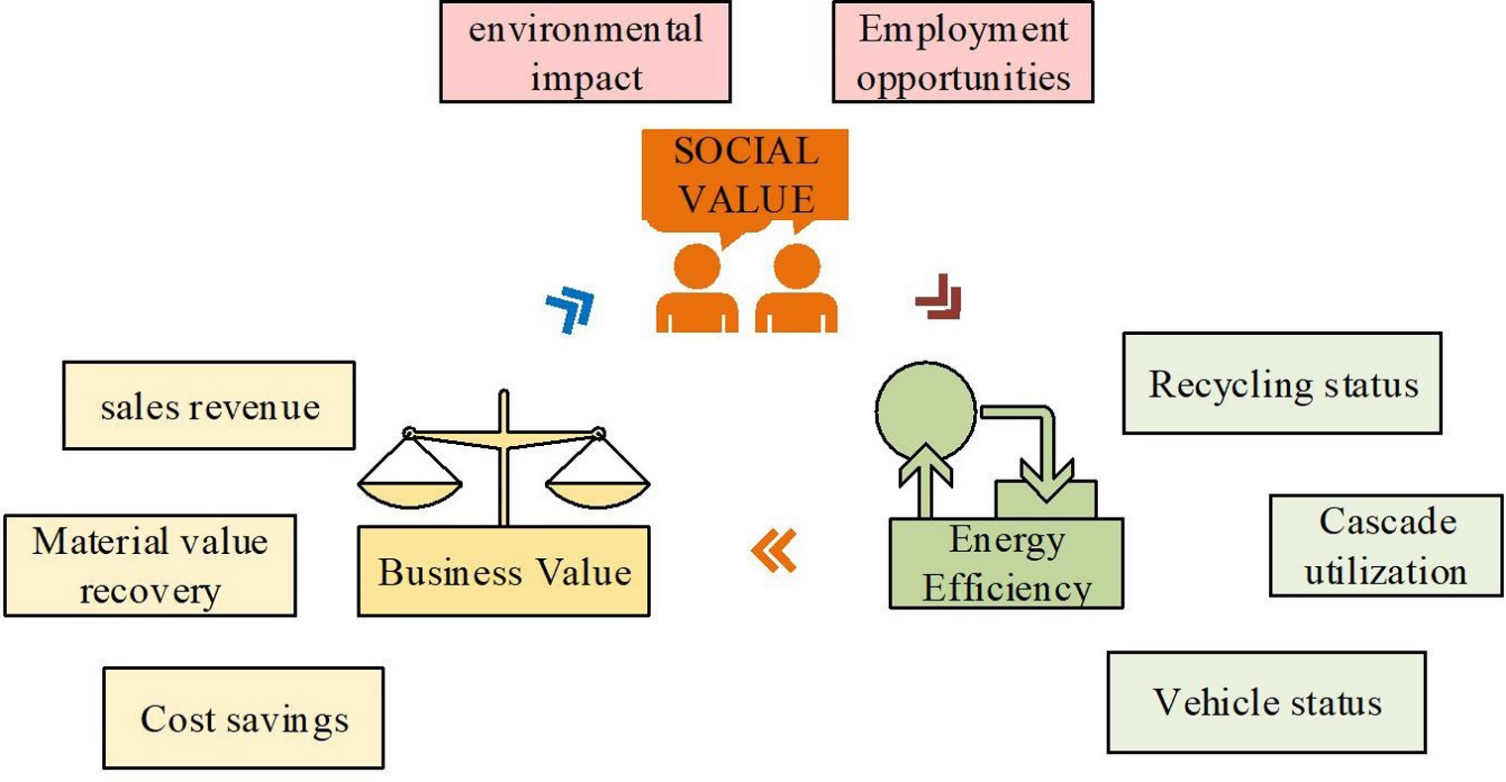
Evaluation system of economic benefits of LBR.

By analyzing and modeling data from fresh and recycled lithium batteries, ML models can predict the remaining capacity of recycled batteries, providing guidance for the reuse of recycled batteries. This prediction capability helps optimize the secondary battery utilization process, reduce the use of unqualified batteries, reduce production costs, and predict the economic benefits of LBR. Before establishing the ML model, it is necessary to establish a reasonable economic benefit assessment system for LBR. The following elements can be considered. The first thing to consider is social value, including both environmental impact and employment opportunities. In terms of the environment, lithium batteries recycling can lessen the negative influence of waste on the environment and save energy resources [23]. In addition, the development of LBR industry has also created employment opportunities for the society. Secondly, it is also necessary to consider commercial value, which includes sales revenue, cost savings, and material value recovery. By lithium batteries recycling, sales revenue can be generated and the cost of producing new batteries can be saved. In addition, valuable materials in recycled lithium batteries can be reused to improve resource recovery rates. Finally, energy benefits need to be considered, which can be divided into three states: recycling, cascade utilization and vehicle use states. In the recycling state, by lithium batteries recycling, energy waste and environmental pollution can be avoided. Cascade utilization refers to the usage of recycled lithium batteries as secondary energy storage devices or other purposes, extending their service life and improving resource utilization efficiency. The state of vehicle use refers to the application of recycled lithium batteries in vehicles such as electric vehicles to achieve clean energy substitution for traditional fuels, thereby reducing carbon emissions and environmental pollution. Through comprehensive consideration of the above factors, a sound evaluation system of economic benefits of LBR can be established to offer reference and guidance for the development of related industries, as shown in Fig 3.

In addition, net present value (NPV) and cost benefit ratio (CBR) are used in the experiment to evaluate the economic feasibility and potential profit of LBR. The NPV of the recycled battery is shown in Eq (1).


$$NPV=\sum \left(\frac{Ct}{1+r}\wedge t\right)-C_{0}$$ (1)

In Eq (1), *Ct* means the cash inflow or cash outflow of each period; *r* denotes the bank rate; *t* expresses the number of time periods; *C*_0_ denotes the initial investment. The ratio between the total benefit and total cost of the project calculated by CBR is shown in Eq (2).


$$CBR=\frac{R}{C}$$ (2)

In Eq (2), *R* expresses total revenue, and *C* represents total cost.

### 3.2 Economic benefit evaluation model based on BP

**Fig 4:**
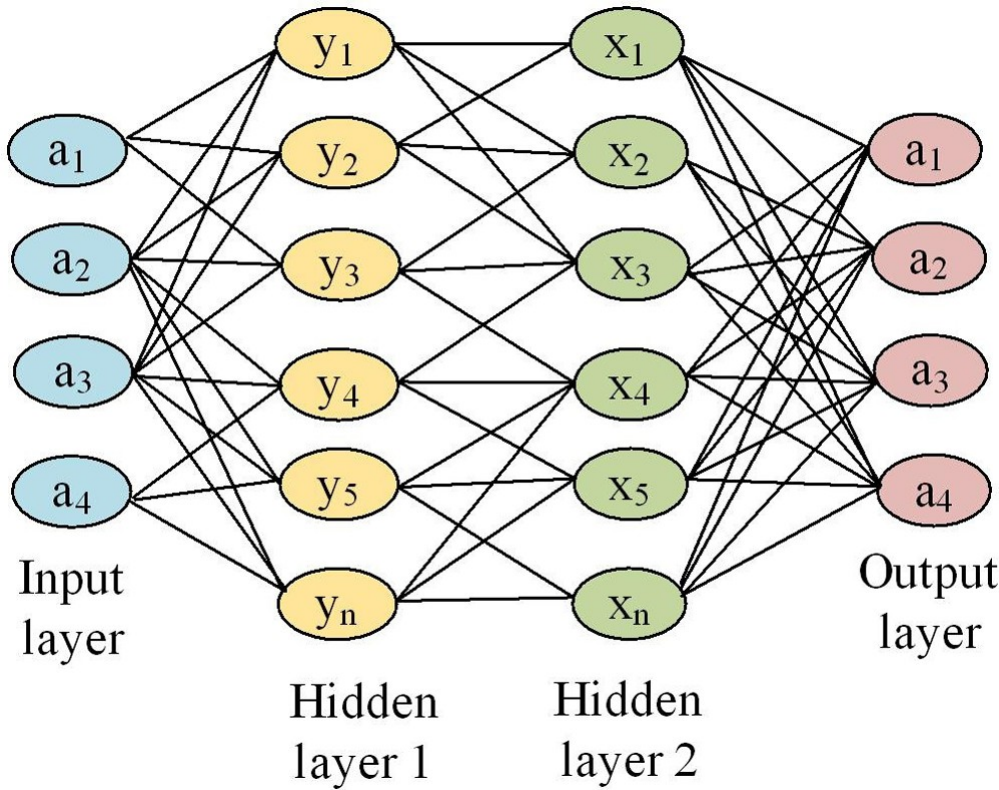
Schematic diagram of BP.

In the field of deep learning, there are many strong neural networks that can be applied to economic benefit evaluation models, such as the Transformer model, but they are often seen as "black boxes" and difficult to explain their internal working mechanisms. In contrast, BP neural networks (especially shallow networks) are easier to understand, and their weights and bias values can provide some explanatory power, helping to understand the importance of the model for different features, which is more suitable for economic benefit evaluation models. Considering the use of BP neural network in this study. It is a multi-layer feedforward neural network. BP includes input layer, hidden layer, and output layer. The input layer receives raw feature data as input, uses hidden layers for feature extraction and non-linear mapping, and the output layer provides the final prediction result, as shown in Fig 4.

**Fig 5:**
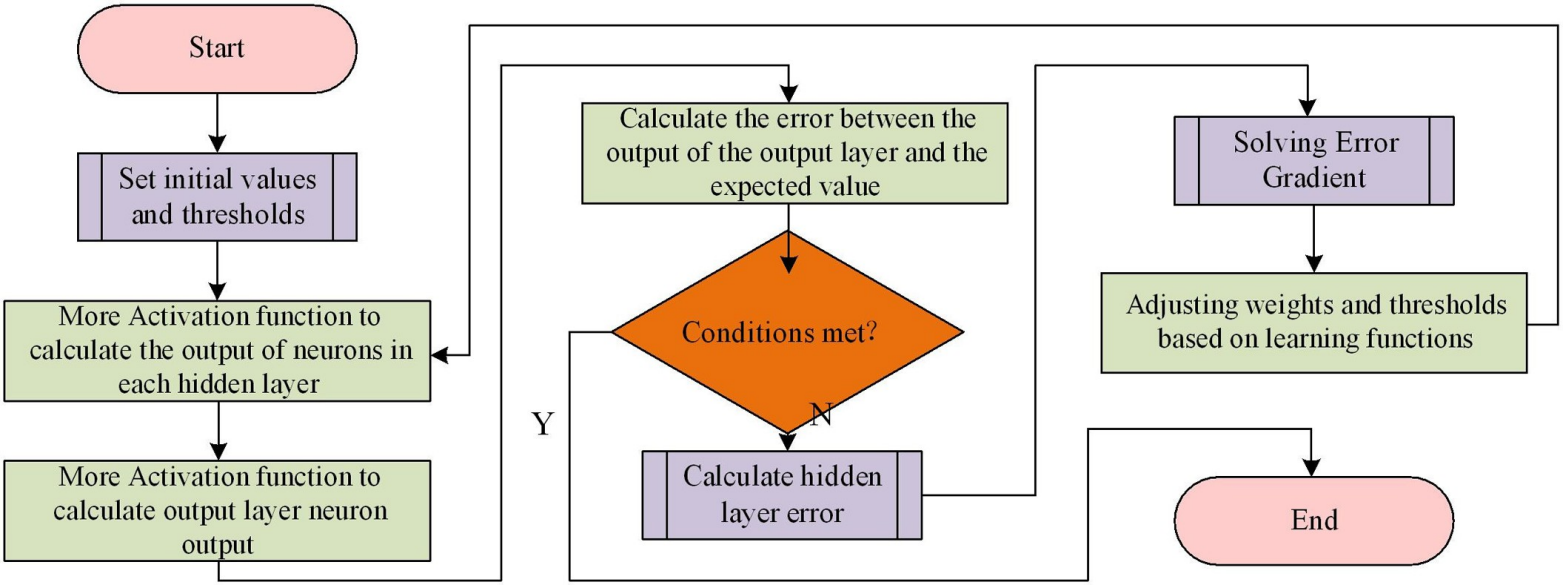
BP neural network algorithm process.

The BP calculates the output results through forward propagation, and the input data is transformed linearly and nonlinearly through each layer of the network until it reaches the output layer, generating prediction results. Each neuron applies a activation function to introduce nonlinear properties. BP uses backpropagating for training and optimization. It is based on the error between the target value and the network output, and updates the weights and biases in the network by calculating gradients and backpropagating the error along the network to reduce the error. Generally, mean squared error (MSE) is used as the loss function of BP. It calculates the gradient of the weight according to it, and optimizes the network through gradient descent. The training of BP includes forward propagation, backpropagation, and parameter update. By iterating multiple times and continuously adjusting the weight and bias of the network, the output of the network approximates the real label, improving prediction accuracy. BP has some important hyperparameters. Research has set the number of hidden layers to 5, the learning rate to 0.001, the number of training iterations to 500, and the attenuation weight to 0.01. Optimizing these hyperparameters is key to achieving better model performance. After the training is completed, a test set needs to be used to evaluate blood pressure. By calculating the error indicators (such as MSE and mean absolute error) between the predicted results and the actual labels, the performance and generalization ability of the model can be evaluated. The algorithm process is shown in Fig 5.

Data preprocessing: Before inputting data, it is necessary to normalize sample data with different ranges, so that the data values are between 0 and 1, making it convenient for the network model to analyze the data. The normalized calculation expression is denoted in Eq (3).


$$y_{i}=\frac{x_{i}-\min(x)}{\max(x)-\min(x)}$$ (3)

In Eq (3), *x*_*i*_ denotes the value of row *i* th data in each column; min(*x*) expresses the maximum value in this column; max(*x*) indicates the minimum value in the same column. Then there is the process of information forward propagation, which involves learning the sample input model, as shown in Eq (4).


$$p^{k}=(x_{1}^{k},x_{2}^{k},\ldots ,x_{n}^{k})$$ (4)

In Eq (4), *p*^*k*^ refers to the input dataset of sample *k*, while $x_{i}^{k}(i=1,2,‥n)$ means the input layer neuron *i* in sample *k*. The output values of each layer of neurons in sequence are calculated using input sample data, connection weights, and thresholds, as shown in Eq (5).


$$\left\{\begin{array}{c} Y_{j}=f(\sum_{i=1}^{n}W_{ij}X_{i}-\theta_{j})\\ Z_{m}=f(\sum_{m=1}^{n}W_{mj}X_{j}-\theta_{m}) \end{array}\right.$$ (5)

In Eq (5), *Y*_*j*_ denotes the hidden layer node of the network; *Y*_*j*_ means the output layer node of the network; *f* function is the activation function of neurons, and sigmaid function is used in the study, as expressed in Eq (6).


$$f(x)=\frac{1}{1+e^{-x}}$$ (6)

In Eq (6), *e* is the natural base, and the third step is the error backpropagation process. The learning error of output layer neurons is shown in Eq (7).


$$\varphi =-(T_{m}-Z_{m})\times f^{\prime }(\sum_{j=1}^{n}W_{mj}X_{j}-\theta_{m})$$ (7)

In Eq (7), *T*_*m*_ expresses the expected output of the output layer node. The learning error of hidden layer neurons is shown in Eq (8)

As shown in.


$$\Phi =f^{\prime }(\sum_{i=1}^{n}W_{ij}X_{i}-\theta_{j})\times \sum_{m=1}^{n}\varphi V_{mj}$$ (8)

In Eq (8), *f*’ function is the derivative function of neuron activation function. The study uses the tanh function as the activation function, and its expression is shown in Eq (9).


$$\tanh(x)=\frac{e^{x}-e^{-x}}{e^{x}+e^{-x}}$$ (9)

In Eq (9), *e* is the natural base and *x* is the input component. Step 4: It needs to determine whether the model error meets the requirements. The standard MSE function is used to calculate the relative error of the model, and the calculation is denoted in Eq (10).


$$E=\sum \frac{1}{2}{\left(T_{m}-Z_{m}\right)}^{2}$$ (10)

In Eq (10), if *E* is less than the given learning accuracy, it indicates that the trained model can achieve the expected error range. Conversely, it is necessary to adjust the weights of each layer of the model. The fifth step is to adjust the weights and thresholds of each layer of the model. The weight adjustment method between the hidden and the output layers is indicated in Eq (11).


$$\left\{\begin{array}{c} V_{mj}(k+1)=V_{mj}(k)+\Delta V_{mj}\\ \theta_{m}(k+1)=\theta_{m}(k)+\eta \varphi \end{array}\right.$$ (11)

In Eq (11), *η* denotes the learning rate of the model. Step 6, it uses new weights and thresholds for forward propagation and error backpropagation processes. Finally, it saves the training network. When it needs to calculate the economic benefits of LBR, it only needs to use the saved model to train and learn the existing data, and then the evaluation results can be directly output.

### 3.3 SR-BPNN

Multicollinearity refers to the high correlation between independent variables in regression analysis [24]. It can lead to instability and decreased explanatory power of the model, making the estimated regression coefficients unreliable or difficult to explain. SR is a common feature selection method, which can be used to reduce the impact of multicollinearity on regression models. Therefore, before using the BP model to evaluate the enterprise value, the SR method is introduced to solve the multicollinearity problem existing among the indicators to determine the optimal self variable quantum set. SR is a method of gradually adding and removing independent variables for constructing predictive models. Preliminary establishment of a multiple regression equation is shown in Eq (12).


$$V=\beta_{0}+\beta_{1}a_{1}+\beta_{2}a_{2}+\cdots +\beta_{n}a_{n}+\varepsilon_{i}$$ (12)

In Eq (12), *β*_*n*_ refers to the estimation coefficient, and *β*_0_ is the intercept term. Based on the results of multiple regression, it needs to identify the variable with the strongest explanatory power for the recovery value among all value drivers, and establish a univariate regression model with the recovery value V, as shown in Eq (13).


$$V=\beta_{01}+\beta_{11}a_{0}+\varepsilon_{1}$$ (13)

In Eq (13), *a*_0_ expresses the variable with the strongest explanatory power. Then, other explanatory variables representing the driving factors of recycling value one by one are added to the established univariate regression model, and least squares regression is performed on them. The regression model in this process is shown in Eq (14).


$$\left\{\begin{array}{c} V=\beta_{02}+\beta_{12}a_{1}+\varepsilon_{2}\\ V=\beta_{03}+\beta_{13}a_{1}+\varepsilon_{3}\\ V=\beta_{04}+\beta_{14}a_{1}+\varepsilon_{4} \end{array}\right.$$ (14)

**Fig 6:**
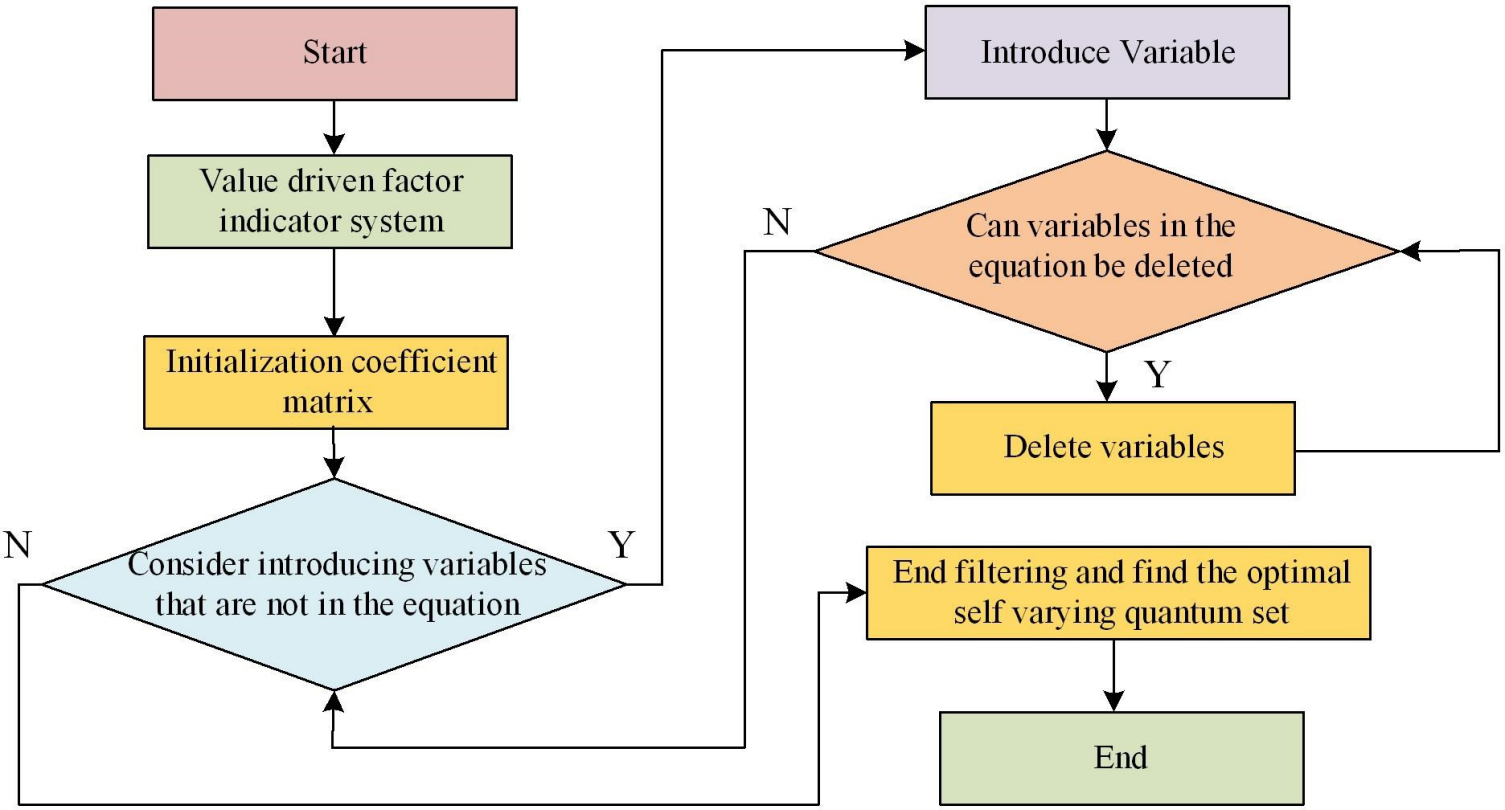
SR algorithm flowchart.

In Eq (14), ε_*i*_ is a random disturbance term. After repeated screening, the variables finally retained in the regression equation are all variables that have a significant impact on the value of LBR recovery. These variables constitute the optimal input variable subset of the BP model. The SR algorithm flow is shown in Fig 6.

**Fig 7:**
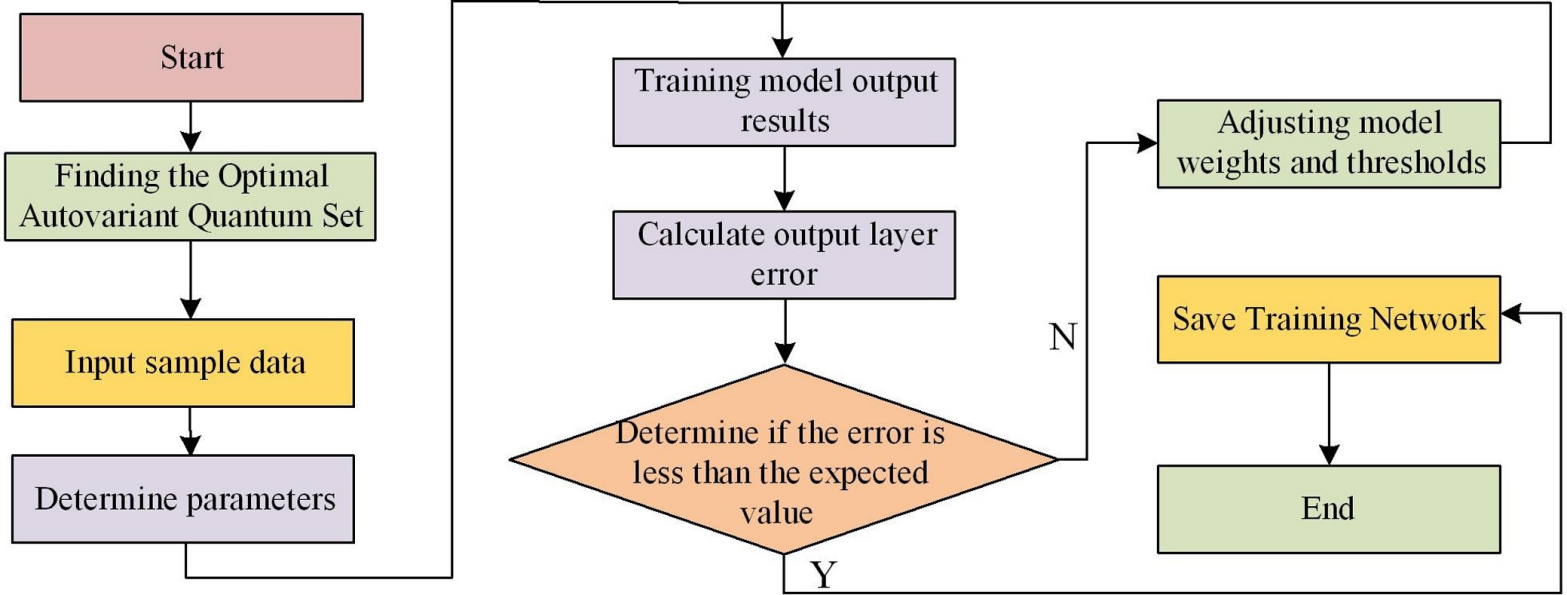
SR-BPNN algorithm process.

The SR-BPNN has the following advantages compared to traditional BP. The first point is that the SR-BPNN uses a SR method to select the most relevant features during the training process and gradually introduces them into the model. This can reduce the impact of redundant features, improve the explanatory power and generalization performance of the model. Secondly, since SR only selects one feature at a time to add to the model, the results of the model are easier to understand and explain. The contribution of each feature and their impact on the predicted results can be gradually observed. Thirdly, by gradually selecting features, the model has lower complexity and requires fewer parameters to be calculated and updated. This may lead to faster training speed and higher computational efficiency. Fourthly, SR-BPNN can balance variables and gradually add them based on their importance. This makes the model somewhat robust to noise and redundant features in the input data. The main steps of the SR-BPNN are shown in Fig 7.

In addition to the process shown in the figure, the complete model building should also include the following processes. Data collection: It collects data related to battery recycling, including battery type, recycling quantity, recycling cost, value after recycling, etc. These data can be obtained through internal enterprise records or industry statistical data. Data pre-processing means cleaning and converting the collected data, including removing outlier, processing missing data, and dividing the data into training and test sets. Feature selection is a crucial step in building predictive models, which selects features that have a significant impact on the economic benefits of battery recycling based on experience and professional knowledge, such as recycling costs and battery types. The study utilizes the SR method to construct the optimal model by gradually adding and removing variables. In this process, the first step is to establish a model based on the single variable with the strongest explanatory power, and then gradually introduce other variables, each time introducing the variable that contributes the most to the model under the condition of controlling for other variables. At the same time, after adding new variables at each step, the significance of existing variables in the model will be re evaluated, and insignificant variables will be promptly removed. This process will be repeated continuously until all variables in the model reach a significant level, ensuring the simplicity and prediction accuracy of the model.

## 4. Model performance analysis and capability evaluation

This chapter is divided into two sub-sections. The first section mainly introduces three algorithms similar to SR-BPNN for horizontal comparison to verify the accuracy and generalization ability of each algorithm in the field of EBA. The second section selects 7 factors such as NPV and CBR as input variables for the neural network model, and evaluates the accuracy and application effectiveness of the SR-BPNN model using evaluation methods such as R^2^ and absolute error (AE).

### 4.1 Horizontal comparison and generalization ability verification of SR-BPNN algorithm

**Fig 8:**
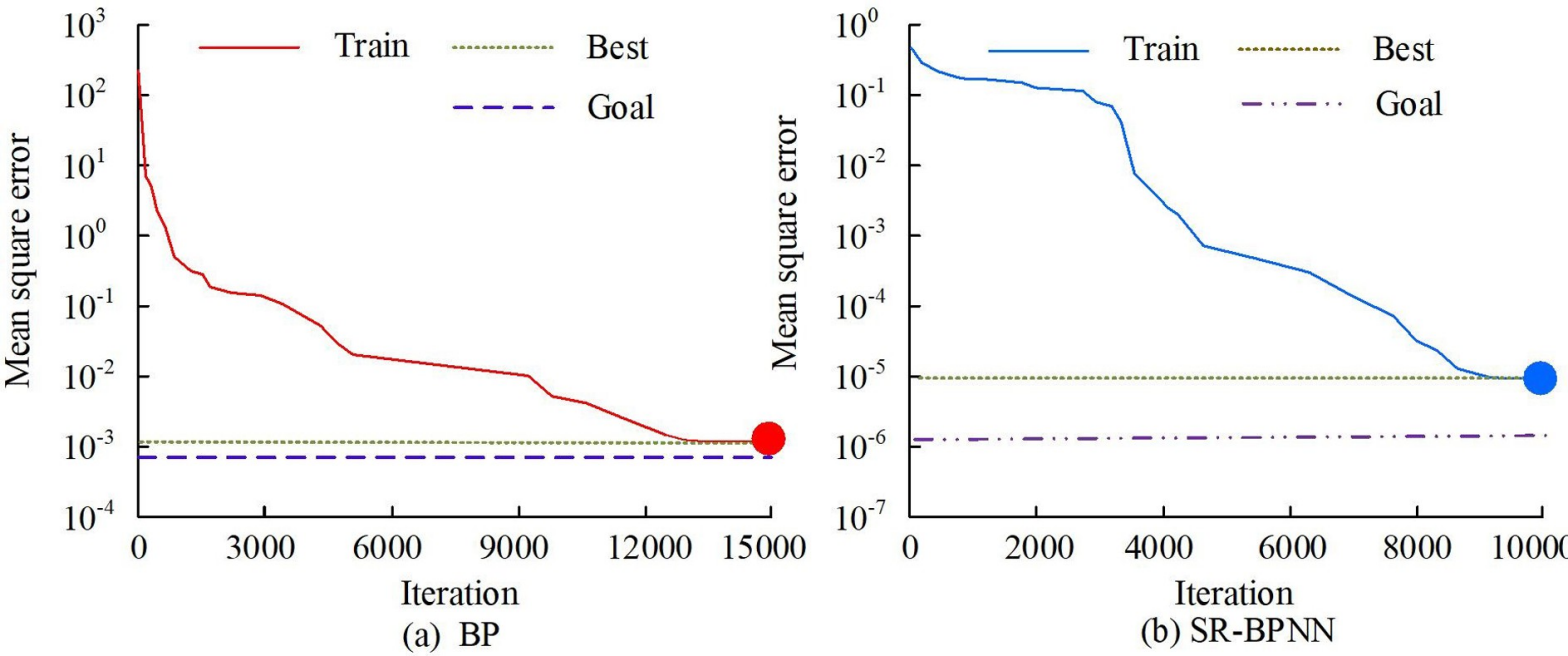
Comparison results of MSE between SR-BPNN and BP algorithms.

The necessity of horizontal comparison of algorithms lied in determining the optimal algorithm selection. Different algorithms might have different time complexity, space complexity, and different performance in different scenarios. By comparing multiple algorithms horizontally, one could better understand their characteristics and applicability, and chose the algorithm that was most suitable for a specific problem. This helped improve program efficiency, reduce resource consumption, and provide a better user experience. Therefore, horizontal comparison of algorithms was an important step in evaluating and selection algorithm. The experiment first verified the performance optimization amplitude of SR-BPNN compared to BP. The obtained results are shown in Fig 8.

**Fig 9:**
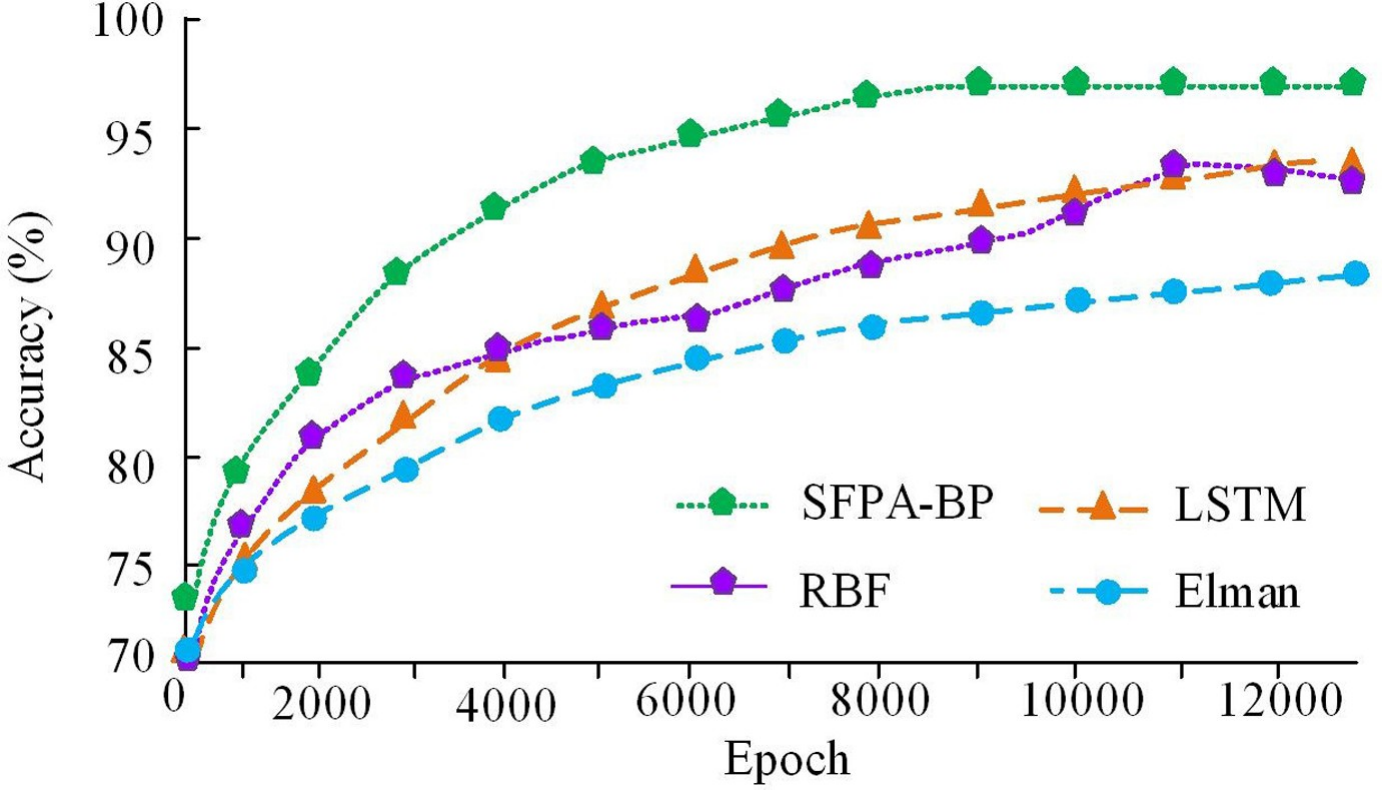
Accuracy curves of various algorithms.

In Fig 8, the horizontal axis represents the iteration times, and the vertical axis denotes the MSE. From Fig 8(A), the speed of BP algorithm gradually slowed down from the 9000th to the 12000th iterations, and converged after the 12000th generation. At this time, the MSE converged between 10–3 and 10–2, which was also the best training performance value of the algorithm. From Fig 8(B), the SR-BPNN algorithm was relatively fast in the 2000–4000 iterations, slowed down in the 6000–8000 generations, and the MSE converged between 10–6 and 10–7. According to the results in Fig 1, compared with GA-BP algorithm, the rate of convergence of SFPA-BP algorithm was increased by about 33%, which showed that this method could effectively improve the convergence accuracy and speed of traditional BP. To further validate the superiority of the SFPA-BP algorithm, the radial basis function neural network (RBF), long short term memory (LSTM), and Elman neural network were introduced for horizontal comparison in the experiment. The experimental outcomes are shown in Fig 9.

**Fig 10:**
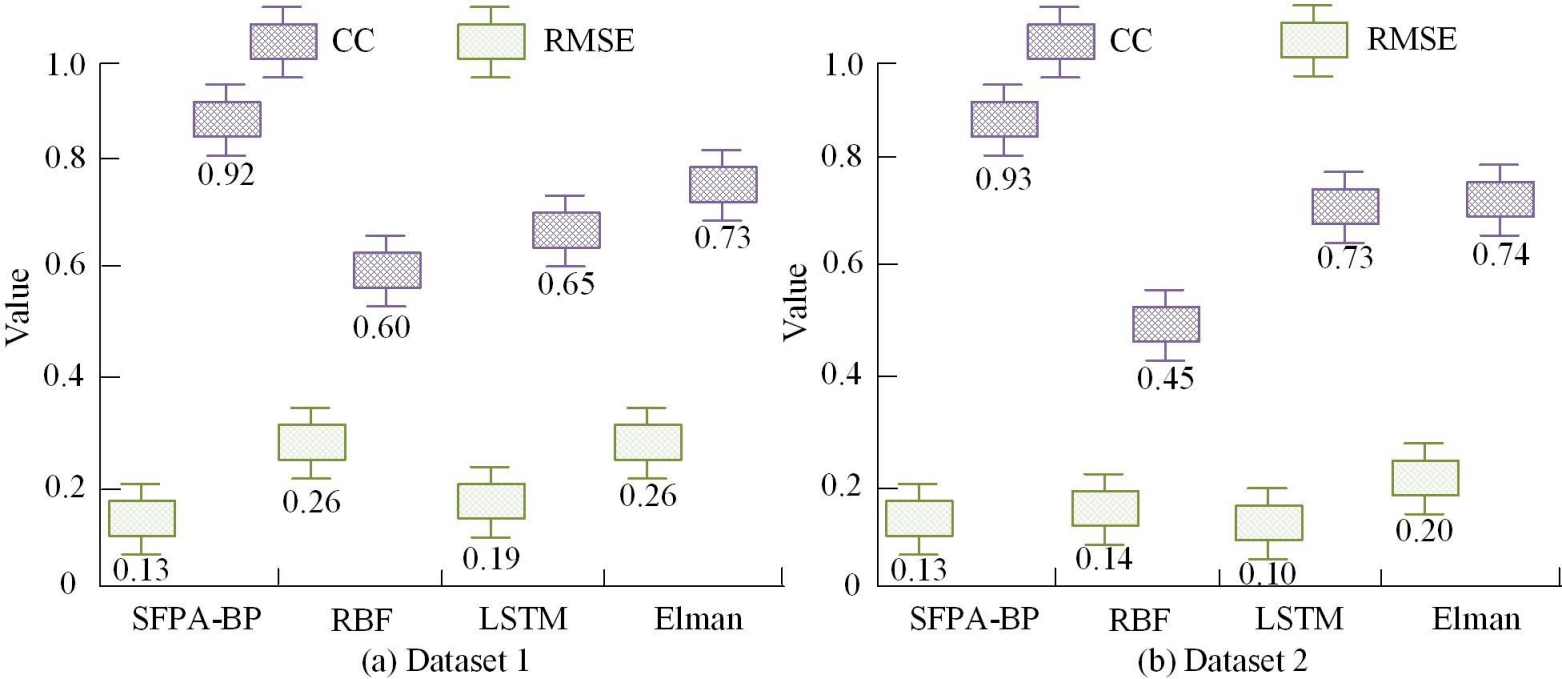
RMSE and CC of each algorithm.

Fig 9 shows the accuracy curve of the four algorithms, SFPA-BP, RBF, LSTM, and Elman, as the iterations increased during the training. From the graph, the SFPA-BP algorithm rapidly increased in accuracy at the beginning of training and tended to converge after 9000 iterations, with the final accuracy curve stabilizing at 96.8%. The LSTM and RBF algorithms also tended to converge after approximately 11000 iterations, with final accuracy converging to 93.2% and 92.8%, respectively. In contrast, the Elman algorithm converged after 12100 iterations and ultimately converged to 86.7%. Elman algorithm had the slowest rate of convergence and the lowest accuracy. In general, SFPA-BP algorithm performed best with fast rate of convergence and high accuracy, while Elman algorithm was relatively poor. These results provided a reference basis for selecting appropriate algorithms. In addition to R2, it was also necessary to select some common measurement methods, including cross correlation coefficient (CC) and root-mean-square deviation (RMSE) coefficient, which can evaluate the similarity between the recovery value predicted by the model and the real value from different aspects. The CC was an indicator that measured the similarity between two variables. By calculating the degree of cross correlation between predicted and actual explicit values, CC could measure the predictive accuracy of the model. A higher CC value meant that the predicted value of the model had a strong correlation with the true value. The RMSE was often used to measure the average difference between the predicted and the real values of the model, calculate the difference between the predicted value and the real value, and square it to find the average root. A lower RMSE value meant that the difference between the model’s predictions and the actual values was small. At the same time, in order to verify the generalization ability of each model, the study obtained business data of nearly 500 lithium battery recycling enterprises from the official database of the China Lithium Battery Association, which was combined into dataset 1. And collected daily operational data from 200 daily necessities recycling enterprises, forming dataset 2. The experiment used dataset 1 and dataset 2 to evaluate the performance of each model. The experimental results are shown in Fig 10.

Fig 10 shows the changes of cross CC and RMSE of SFPA-BP, RBF, LSTM and Elman in datasets 1 and 2. The SFPA-BP model CC and RMSE trained using different datasets had little difference, indicating that the model had strong generalization ability. From Fig 10(A), SFPA-BP had the highest CC value when using dataset 1, indicating a strong correlation between the model’s predictions and the true values. In addition, the RMSE of SFPA-BP was the smallest when dataset 1 was used, which was 0.13, 0.06, and 0.13 lower than RBF, LSTM, and Elman, respectively, indicating that the difference between the predicted value of SFPA-BP and the actual displayed value was the smallest when dataset 1 was used. From Fig 10(B), the accuracy of using RBF and LSTM had significant changes, and the generalization ability of these two models was relatively poor.

### 4.2 Performance evaluation and application effect analysis of SR-BPNN model

Seven factors were selected as the input variables of the neural network model to explore their impact on the economic benefits of Keli battery recycling. These factors included the social value, environmental values, sales revenue, cost savings, material value, NPV and CBR of each gram of LBR. The data came from the business activities and recovery value data published by a LBR enterprise in Shandong province. The result was that data set 1 was obtained after data cleansing and other pre-operations. By using this data, researchers could obtain the actual situation of the enterprise and apply it to the neural network model, with some data shown in Table 1.

**Table 1:** Public recycling value data of a LBR enterprise.

Recycling batch	SOCIAL VALUE($)	Environmental value($)	Sales revenue($)	Cost savings($)	NPV	CBR
R45681	625	856	16848	4861	0.54	1.26
R45682	638	886	16563	8456	-0.15	0.85
R45683	626	896	16862	4562	0.68	1.34
R45684	628	895	16563	8432	0.54	1.24
R45685	924	854	16568	4568	0.33	1.13
R45686	632	433	16886	4931	0.54	1.35
R45687	866	823	16842	5489	0.12	1.43
R45688	683	896	16563	10651	-0.36	0.35
R45689	425	865	16862	8613	0.11	1.16
R45690	629	887	16562	4563	0.56	1.45

**Fig 11:**
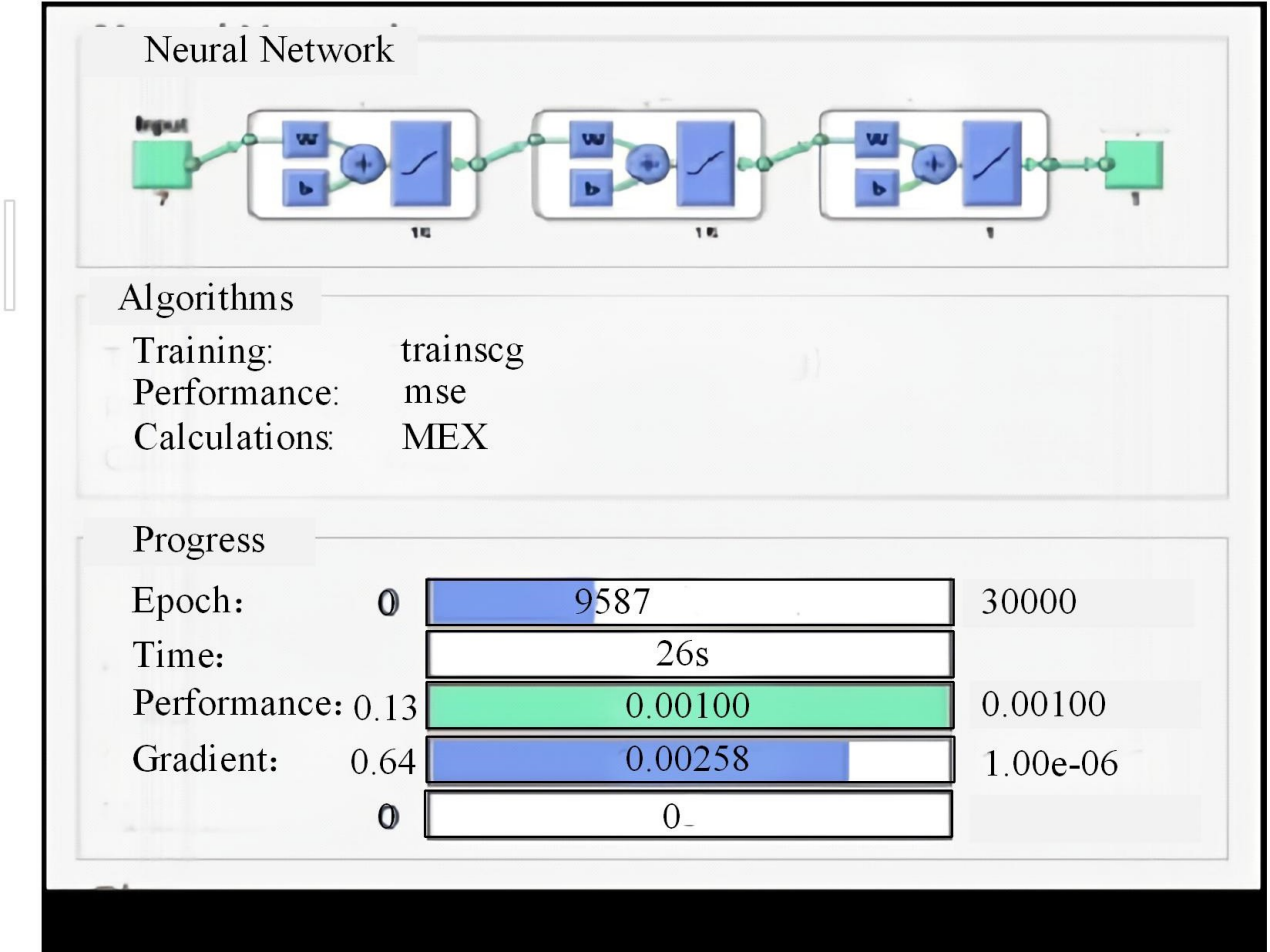
SR-BPNN training results.

From Table 1, in most cases, the NPV of the LBR industry was positive, which meant that the return on investment of the project was higher than the bank rate, and it could be regarded as a profitable investment opportunity. The CBR of most batches was greater than 1, indicating that the economic benefit of the project was higher than the cost, that is, each unit cost generated more than 1 unit of economic return. To evaluate the performance of the SR-BPNN model, 70% of the total sample size was used as training samples, and the remaining 30% was used as testing samples. The training samples were used to establish the model, while the testing samples were used to verify and test the fitting effect of the model. The maximum learning times model.trainParam.epochs = 30000, and the minimum global error model.trainParam.goal = 1e-3. The experimental results are shown in Fig 11.

**Fig 12:**
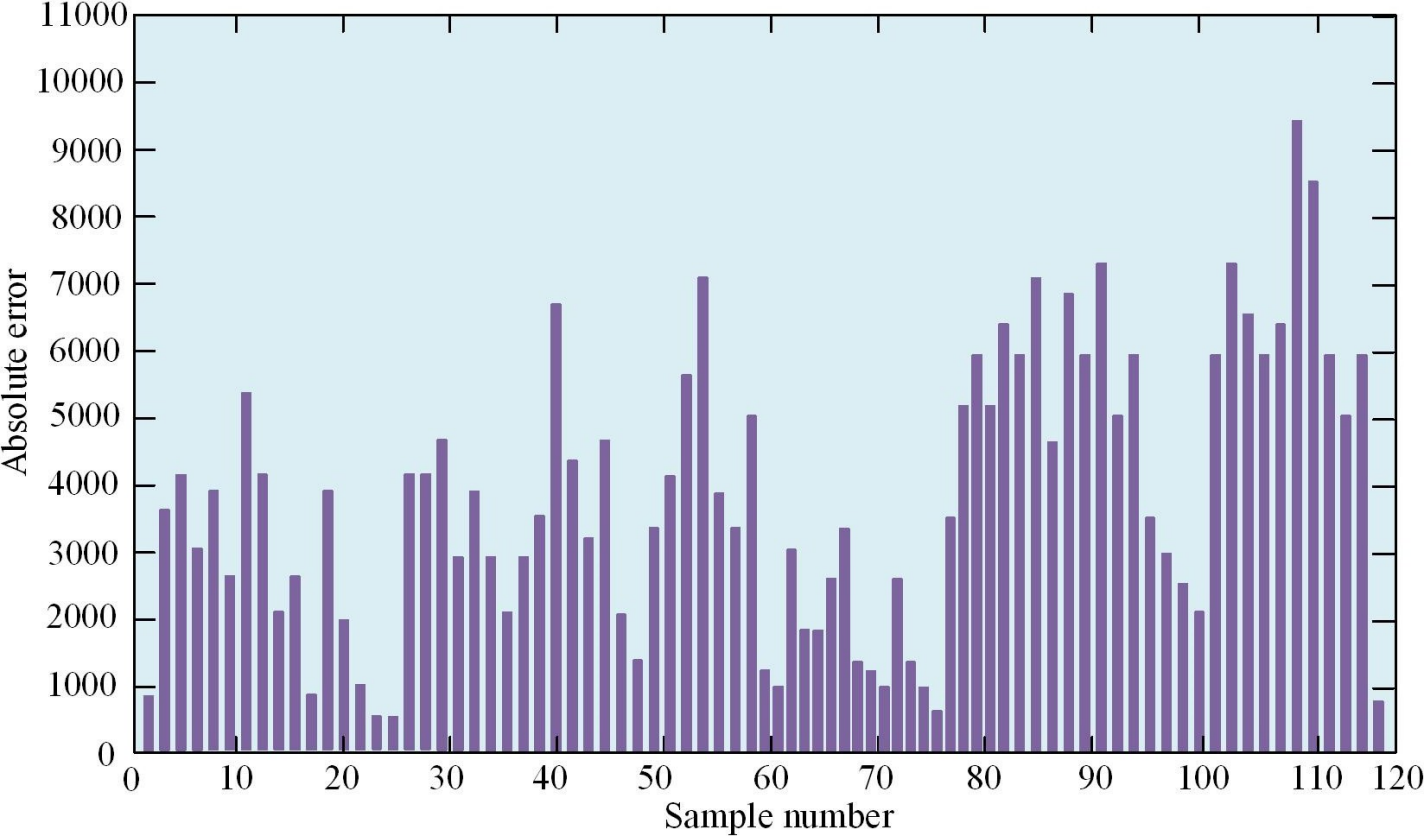
SR-BPNN AE diagram.

Fig 11 shows the training results of SR-BPNN. From it, the total number of iterations of the model has reached 9587, indicating that the model has undergone a large number of iterative steps for parameter adjustment and optimization, indicating that the model has undergone more comprehensive learning and adjustment. But the learning time of the model only took 26 seconds, indicating that the training and learning time of the model was shorter and the running speed was faster. During the training, the gradient value was 0.00258, and a smaller gradient value indicated a smaller degree of parameter adjustment, indicating that the model might be approaching convergence or fine-tuning in a gentle region. Using AE to evaluate the training accuracy of the SR-BPNN model was a common method. AE was the absolute value of the difference between the predicted and the actual values, and the AE of some samples is shown in Fig 12.

**Fig 13:**
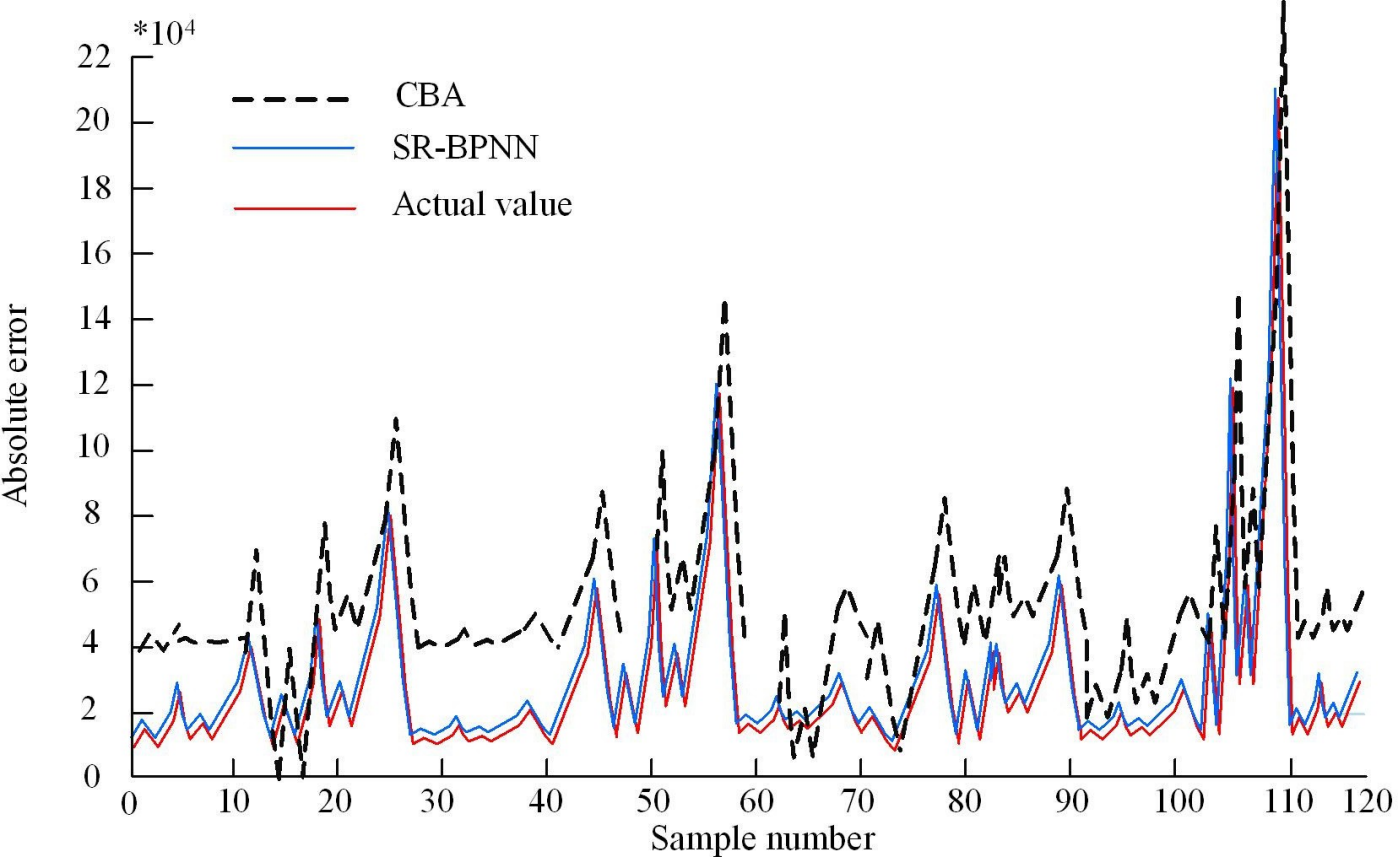
Comparison between predicted and actual values.

Fig 12 shows the AE statistics of some training samples. Observing the Figure, there was a significant AE between the predicted and the true values of only a few samples. Considering the scale difference between LBR batches, these large AEs might be caused by the high value of some recycling batches. Since LBR batches might have different economic benefit ranges, some high-value recycling batches might lead to large prediction errors. However, the large AE of these individual samples alone could not indicate that the model was unreliable. To analyze the application effect of the model, the Cost Benefit Analysis (CBA) model proposed in reference [25] was introduced for comparison. The SR-BPNN model and CBA were applied to the actual operation of enterprises, and the predicted values of the model were used to determine whether each batch of lithium batteries was worth recycling. The actual economic benefits of each batch of LBR were compared with the predicted values of the SR-BPNN model. This comparative analysis provides decision-making basis for enterprises, which can optimize recycling strategies and improve economic benefits. The comparison between the actual economic returns of each sample in the dataset and the predicted values of SR-BPNN is shown in Fig 13.

**Fig 14:**
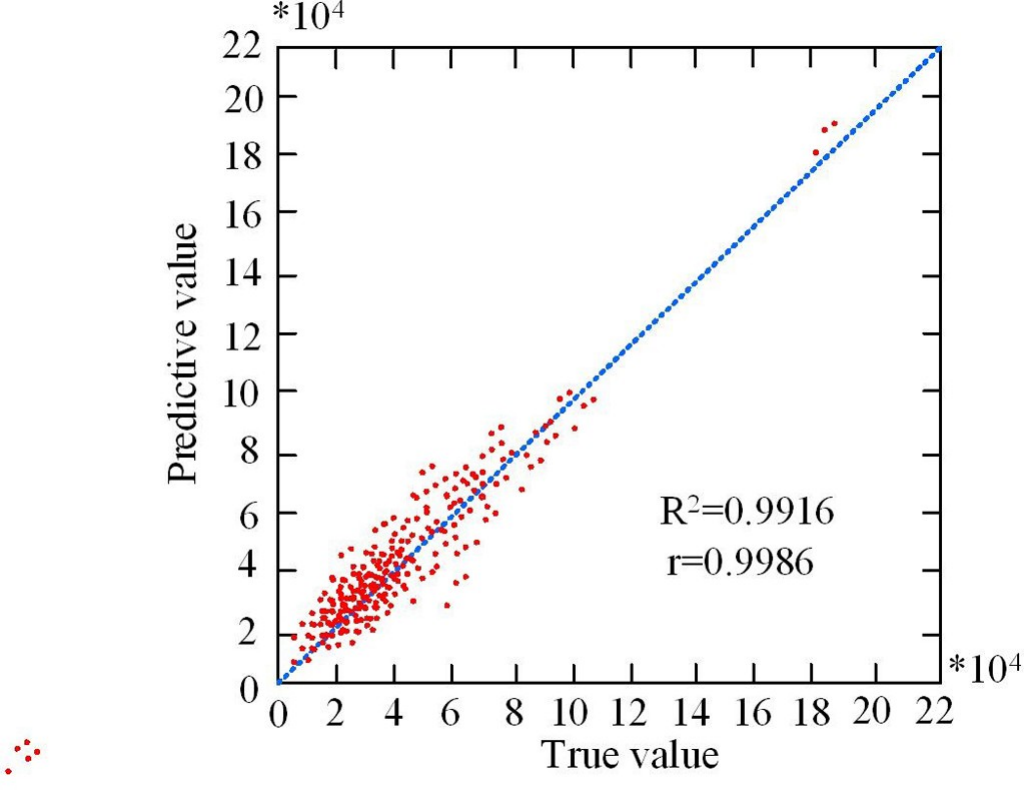
Scatter fitting plot.

In Fig 13, the red curve represents the SR-BPNN predicted economic benefits of each batch of LBR, while the blue curve represents the actual economic benefits of the enterprise recycling this batch of lithium batteries. From this, it can be seen that these two curves basically overlap, indicating that the predicted economic benefits of SR-BPNN for the sample are very close to the actual economic benefits. This means that the model can effectively capture the actual recycling value of each batch of lithium batteries and has high applicability, providing accurate guidance for the actual production activities of enterprises. This consistency can lead us to believe that the model is also applicable to other types of EBA. The error of the CBA model proposed in reference [25] is relatively small, but compared to SR-BPNN, it is much worse. In the experiment, the SR-BPNN model was applied to a garbage recycling enterprise in Sichuan to predict the economic benefits generated by each batch of garbage recycling. Fitting curves and regression lines were used to describe the trend between variables, and display the relationship between variables in the form of a scatter fitting graph. The SR-BPNN scatter fitting graph is shown in Fig 14.

In Fig 14, each data point is represented by horizontal and vertical coordinates, corresponding to two different variables, namely the actual economic benefits generated by each batch of garbage collection and the predicted value of SR-BPNN. These data points are presented in the form of scatter points in the chart, allowing us to intuitively observe their distribution patterns. From the graph, there was a linear correlation between the training results and the true values. The goodness of fit R2 was an indicator that measured the degree of fit, with values ranging from 0 to 1. Here, the R2 value was 0.9916, which was very close to 1, indicating that the model had a very good fitting effect and high accuracy when applied to the field of EBA. In addition, the CC was also an indicator for evaluating the strength of the relationship between two variables, with values ranging from -1 to 1. The closer the absolute value was to 1, the stronger the relationship. In Fig 1, the CC r was 0.9986, which was very close to 1, further supporting the success of BP training. Finally, in order to determine that the conclusions of the study are widely correct, the study repeated 20 experiments, and the results are basically consistent with this experiment. Therefore, it can be determined that the results of this study are correct.

## 5. Conclusion

To solve the problem that it is difficult to accurately evaluate the value of LBR, a model for analyzing the economic benefits of LBR was established by combining SR and BP. The outcomes denoted that the NPV of the LBR industry was positive in most cases, and the CBR of most LBR batches was greater than 1. After experimental verification, the R2 value of the SR-BPNN model was 0.9916, which was very close to 1, indicating the success of the training. In addition, the CC r of the model was 0.9986, which was very close to 1. To compare the SR-BPNN model horizontally, three algorithms, RBF, LSTM, and Elman, were introduced in the experiment and trained separately. The outcomes indicated that the accuracy of the SFPA-BP algorithm converged to 96.8%, while the LSTM and RBF algorithms also tended to converge after about 11000 iterations, with the final accuracy converging to 93.2% and 92.8%, respectively. In contrast, the Elman algorithm converged after 12100 iterations and ultimately converged to 86.7%. At the end of the experiment, the generalization ability of each model was verified, and the findings expressed that the SFPA-BP model CC and RMSE trained with different datasets were not significantly different, indicating that the model had strong generalization ability. SFPA-BP had the highest CC value when using dataset 1, indicating a strong correlation between the model’s predictions and the actual values. In addition, the RMSE of SFPA-BP was the smallest when dataset 1 was used, which was 0.13%, 0.06% and 0.13% lower than RBF, LSTM and Elman respectively, indicating that the difference between the predicted value of SFPA-BP and the actual displayed value was the smallest when dataset 1 was used. However, in the case of using dataset 2, the RMSE of LSTM was 0.10, which was 0.03 lower than the SFPA-BP algorithm, indicating that LSTM was more accurate in the case of using dataset 2. This is also an area that needs improvement in future research.

## References

[1] Danjuma M. U., Yusuf B., Yusuf I.. Reliability, availability, maintainability, and dependability analysis of cold standby series-parallel system. JCCE, vol. 1, no. 4, pp. 193–200. July. 2022, doi: 10.47852/bonviewJCCE2202144

[2] Nimrah S., Saifullah S.. Context-Free Word Importance Scores for Attacking Neural Networks. JCCE, vol. 1, no. (4), pp. 187–192, September. 2022, doi: 10.47852/bonviewJCCE2202406

[3] Ejegwa P. A., Agbetayo J. M.. Similarity-distance decision-making technique and its applications via intuitionistic fuzzy pairs. JCCE, vol. 2, no. 1, pp. 68–74, July. 2022, doi: 10.47852/bonviewJCCE512522514

[4] Song S., Xiong X., Wu X.. Modeling the SOFC by BP neural network algorithm. Int J Hydrogen Energ, vol. 46, no. 38, pp. 20065–20077, June. 2021, doi: 10.1016/j.ijhydene.2021.03.132

[5] Mukkamala R., Yavarimanesh M., Natarajan K., O Hahn J., Kyriakoulis K. G., Avolio A., et al. Evaluation of the accuracy of cuffless blood pressure measurement devices: challenges and proposals. HTN, vol. 78, no. 5, pp. 1161–1167, September. 2021, doi: 10.1161/HYPERTENSIONAHA.121.17747 34510915 PMC8516718

[6] Rahimi K., Bidel Z., Nazarzadeh M., Copland E., Canoy D., Ramakrishnan R., et al. Pharmacological blood pressure lowering for primary and secondary prevention of cardiovascular disease across different levels of blood pressure: an individual participant-level data meta-analysis. Lancet, vol. 397, no. 10285, pp. 1625–1636. May. 2021, doi: 10.1016/S0140-6736(21)00590-0 33933205 PMC8102467

[7] Wright L. G., Onodera T., J Stein M M. M., & Bharath J. N.. Deep physical neural networks trained with backpropagation. NATURE, vol. 601, no. 7894, pp. 549–555, April 2021. 10.1038/s41586-021-04223-6 PMC8791835 35082422

[8] Zhang M., Wang J., Wu J., Belatreche A., Amornpaisannon B., Zhang Z., et al. Rectified linear postsynaptic potential function for backpropagation in deep spiking neural networks. IEEE T Neur Net Lear, vol. 33, no. 5, pp. 1947–1958, May. 2022, doi: 10.1109/TNNLS.2021.3110991 34534091

[9] Aswathy A. L., Hareendran A., SS V. C.. COVID-19 diagnosis and severity detection from CT-images using transfer learning and back propagation neural network. J Infect Public Heal, vol. 14, no. 10, pp. 1435–1445, October 2021, doi: 10.1016/j.jiph.2021.07.015 PMC8319091 34362695

[10] Sindhu J., Namratha R.. Impact of artificial intelligence in chosen Indian commercial bank-a cost benefit analysis. APJM, vol. 10, no. 4, pp. 377–384. February. 2019, doi: 10.5958/2321-5763.2019.00057.X

[11] Ibrahim A., Rundle-Thiele S., Knox K., Almestarihi R. B.. The relative merit of two segmentation approaches: executives views and a cost-benefit analysis. J Soc Market, vol. 12. no. 4, pp. 607–622, July. 2022, doi: 10.1108/JSOCM-01-2022-0026

[12] Cronin P., Stein-Parbury J., Sommer J., Gill K. H.. What about value for money? A cost benefit analysis of the South Eastern Sydney Recovery and Wellbeing College. J Mens Health, vol. 32, no. 1, pp. 63–70, May 2021, doi: 10.1080/09638237.2021.1922625 33966575

[13] Szymanski T., Ashton R., Sekelj S., Petrungaro B., Pollock K. G., Sandler B., et al. Budget impact analysis of a machine learning algorithm to predict high risk of atrial fibrillation among primary care patients. Europace, vol. 24, no. 8, pp. 1240–1247. February. 2022, doi: 10.1093/europace/euac016 35226101

[14] Boltürk E., Haktanır E.. Decomposed fuzzy cost-benefit analysis and an application on ophthalmologic robot selection. Eng Econ, vol. 68, no. 1, pp. 20–33, Feb 2023, doi: 10.1080/0013791X.2023.2179709

[15] Ward Z. J., Scott A. M., Hricak H., Atun R.. Global costs, health benefits, and economic benefits of scaling up treatment and imaging modalities for survival of 11 cancers: a simulation-based analysis. Lancet Oncol, vol. 22, no. 3, pp. 341–350, March, 2021, doi: 10.1016/S1470-2045(20)30750-6 33662286 PMC8033570

[16] Sheikh S. S., Anjum M., Khan M. A., Hassan S. A., Khalid H. A., Gastli A., et al. A battery health monitoring method using machine learning: A Data-Driven Approach. Energies, vol. 13, no. 14, p. 3658, Jul. 2020.

[17] Khalid M., Sheikh S. S., Janjua A. K. and Khalid H. A.. Performance Validation of Electric Vehicle’s Battery Management System under state of charge estimation for lithium-ion Battery. 2018 International Conference on Computing, Electronic and Electrical Engineering (ICE Cube), Quetta, 2018, pp. 1–5, doi: 10.1109/ICECUBE.2018.8610969

[18] Sheikh S.S., Shah F.A., Athar S.O., and Khalid H.A.. A Data-driven comparative analysis of lithium-ion battery state of health and capacity estimation. Electric Power Components and Systems, 2023, pp.1–11

[19] Shah F. A., Shahzad Sheikh S., Mir U. I., and Owais Athar S.. Battery health monitoring for commercialized electric vehicle batteries: Lithium-Ion. 2019 International Conference on Power Generation Systems and Renewable Energy Technologies (PGSRET), Istanbul, Turkey, 2019, pp. 1–6.

[20] Cheng X. H., Liu H., Yuan H., Peng H. J., Tang C.. Huang J. Q., et al. perspective on sustainable energy materials for lithium batteries. SusMat, vol. 1, no. 1, pp. 38–50, March 2021, doi: 10.1002/sus2.4

[21] Deshwal D., Sangwan P., Dahiya N.. Economic analysis of lithium ion battery recycling in India. WPC, vol. 124, no. 4, pp. 3263–3286, January 2022.

[22] Mao J., Ye C., Zhang S., Xie F, Zeng R., Davey K., et al. Toward practical lithium-ion battery recycling: adding value, tackling circularity and recycling-oriented design. Energ Environ Sci, vol. 15, no. 7, pp. 2732–2752, May. 2022, doi: 10.1039/D2EE00162D

[23] Rajaeifar M. A., Raugei M., Steubing B.. Life cycle assessment of lithium‐ion battery recycling using pyrometallurgical technologies. J Ind Ecol, vol. 25, no. 6, pp. 1560–1571, June. 2021, doi: 10.1111/jiec.13157

[24] Uzuke C. A., Ezeilo I. C.. On identifying influential observations in the presence of multicollinearity. OJS, vol. 11, no. 2, pp. 290–302, March. 2021, doi: 10.4236/ojs.2021.112016

[25] Singh J. and Tiwari R.. Cost benefit analysis for V2G implementation of electric vehicles in distribution system. IEEE T Ind Appl, vol. 56, no. 5, pp. 5963–5973, Sept.-Oct. 2020, doi: 10.1109/TIA.2020.2986185

